# Controlled Delivery of Human Cells by Temperature Responsive Microcapsules

**DOI:** 10.3390/jfb6020439

**Published:** 2015-06-18

**Authors:** W.C. Mak, K. Olesen, P. Sivlér, C.J. Lee, I. Moreno-Jimenez, J. Edin, D. Courtman, M. Skog, M. Griffith

**Affiliations:** 1 Integrative Regenerative Medicine Centre, Department of Clinical and Experimental Medicine, Linköping University, SE58185, Linköping, Sweden; E-Mails: kim.olesen@liu.se (K.O.); petter.sivler@s2m.se (P.S.); chyanjang@gmail.com (C.J.L.); inesmorenojimenez@gmail.com (I.M.-J.); joel.edin@liu.se (J.E.); marten.skog@liu.se (M.S.); may.griffith@liu.se (M.G.); 2 Biosensors and Bioelectronics Centre, Department of Physics, Chemistry and Biology, Linkӧping University, SE58183, Linköping, Sweden; 3 Bone & Joint Research Group, Stem Cells & Regeneration Institute of Developmental Sciences, Southampton General Hospital, Southampton, Hampshire SO16 6YD, UK; 4 Regenerative Medicine Program, Ottawa Hospital Research Institute, Ottawa, Ontario K1H 8L6, Canada; E-Mail: dcourtman@ohri.ca

**Keywords:** cell encapsulation, microcapsules, hydrogel, cell delivery, temperature responsive, human fibroblast, human umbilical vein endothelial cells

## Abstract

Cell therapy is one of the most promising areas within regenerative medicine. However, its full potential is limited by the rapid loss of introduced therapeutic cells before their full effects can be exploited, due in part to anoikis, and in part to the adverse environments often found within the pathologic tissues that the cells have been grafted into. Encapsulation of individual cells has been proposed as a means of increasing cell viability. In this study, we developed a facile, high throughput method for creating temperature responsive microcapsules comprising agarose, gelatin and fibrinogen for delivery and subsequent controlled release of cells. We verified the hypothesis that composite capsules combining agarose and gelatin, which possess different phase transition temperatures from solid to liquid, facilitated the destabilization of the capsules for cell release. Cell encapsulation and controlled release was demonstrated using human fibroblasts as model cells, as well as a therapeutically relevant cell line—human umbilical vein endothelial cells (HUVECs). While such temperature responsive cell microcapsules promise effective, controlled release of potential therapeutic cells at physiological temperatures, further work will be needed to augment the composition of the microcapsules and optimize the numbers of cells per capsule prior to clinical evaluation.

## 1. Introduction

Cell-based therapies are now being evaluated for their potential to repair and regenerate damaged or otherwise failing organs from almost all parts of the body. However, despite promising initial results, in many cases, the outcomes are sub-optimal, due to the massive and rapid loss of grafted cells before they can exert their full desired therapeutic effects. One source of cell loss is from anoikis, the massive programmed cell death that occurs when adherent cells are removed from their substrates for transplantation [1]. It is believed that the lack of extracellular matrix (ECM) cues due to the disruption of matrix attachment is the trigger for the apoptosis that occurs. When cells are grafted into pathological tissue after damage from disease or injury, they are essentially introduced into a hostile environment. The tissue may be inflamed, or the healthy and normally pliable tissue may have been replaced by scar tissue that lacks innervation or a vascular supply. This makes it difficult for newly introduced cells to remain viable and to stably integrate into the damaged area. Hence, there are numerous examples of cell-based therapies that initially perform well but decline as introduced cells are lost, confirming the need for a controlled differentiation environment for therapeutic progenitor cells or stem cells [2,3].

In an attempt to circumvent anoikis during the delivery of therapeutic mesenchymal stromal (stem) cells (MSCs), Courtman *et al*. transiently encapsulated single cells, each within a “cocoon” of agarose that included the ECM components, fibrinogen and fibronectin [4]. The ECM components provided the missing cell-substratum cues during the grafting process that were needed to remain viable. The encapsulated cells showed a higher expression of survival genes, Akt/P13K and MAPK/ERK, with overall higher survival rates when injected into an ischemic muscle model. More recently, this single cell encapsulation method was tested with cardiac stem cells with improvement in cell survival after injection into mouse myocardial infarction models [5].

The high impact of the extracellular environment controlling cellular function is well known [5,6,7,8,9]. The biomaterial that is used to encapsulate cells will define the extracellular environment for the cells. In most cell encapsulation methods, hydrogels are used to mimic the hydrated native ECM properties to allow for diffusion of solutes, as well as to reduce the friction between the cells and their surrounding tissue [10,11,12,13,14]. Obtaining capsules of uniform shape and size around single cells is a challenge, making reproducibility problematic [15]. The size of the capsules is essential for the function and the micro-sized ones are preferred because they will facilitate a higher diffusion rate and thereby improve cell viability [16,17].

A wide range of biomaterials that include synthetic polymers, polysaccharides (such as agarose, alginate and chitin) and naturally-derived ECM proteins (such as collagen, fibrinogen and gelatin) have been investigated for cell encapsulation [18]. The seaweed-derived polysaccharides, alginate and agarose are in particular very suitable for producing compact coatings. Neither has been known to cause any significant immunoreaction when introduced into the human body, showing that they are at minimum, immunologically equivalent to naturally derived ECM proteins [19]. In fact, a three-year study of agarose as a filler material for lips has shown that it was less immunoreactive than hyaluronic acid or collagen [20]. Agarose has thermo-gelling properties that can provide structural integrity when making micro-sized capsules or beads. The thermo-gelling properties facilitate the process of making beads and harvesting them without the use of chemicals, compared to alginate where chemical cross-linking is needed [21]. Alginate cross-linked with calcium and coated with poly(L-lysine) (PLL) has been tested for use in immunoisolation for xenogeneic transplantation, e.g. of islets of Langerhans containing beta-cells for insulin production in diabetes treatment [22]. Beads of agarose, however, have failed to work as immunoisolation barriers, mainly because they are generally more porous. However, the higher porosity lends itself to use for temporary encapsulation situations where it is desirable for the cells to be more easily released into the environment.

Agarose has been found to be very useful in methods like electrophoresis because of the low amount of charges in the gel, which gives accurate results without internal interruption. Pure agarose hydrogels have therefore successfully been used as a non-adhesive model for studying anoikis [23]. As a cell encapsulation agent, agarose will therefore not be able to allow for attachment and up-regulation of the transmembrane receptors that are needed for cell–cell and cell–substratum interactions once the cells are released into their target sites. To improve cell viability and circumvent anoikis, the microcapsules therefore also need to contain either ionic charges or fine fibrillary structures within the matrix that can be used for cell attachment. As attachment points are not provided by agarose, these can be provided by naturally derived ECM proteins [24]. 

Collagen is the major component of the mammalian ECM and is an exceptionally suitable biomaterial for cell attachment [25]. However, collagen is a large macromolecule that is difficult to manipulate. Collagen is also temperature sensitive and cannot be autoclaved, in contrast to agarose, alginate and gelatin. Gelatin is denatured collagen, comprising smaller fragments of the same material. Gelatin has many “cell-friendly” properties and like collagen, can be used to improve cell adhesion. However, it is only possible to encapsulate cells using gelatin and/or collagen alone, if the proteins are chemically cross-linked. Unfortunately most cross-linkers for these proteins are cytotoxic. Only a few possible methods exist where the cells survive the cross-linking but none of them are completely cell friendly [26]. Fibrinogen is a large glycoprotein (Mw 340,000) and a dimer comprising three pairs of non-identical chains, Aα, Bβ and γ. 

**Figure 1:**
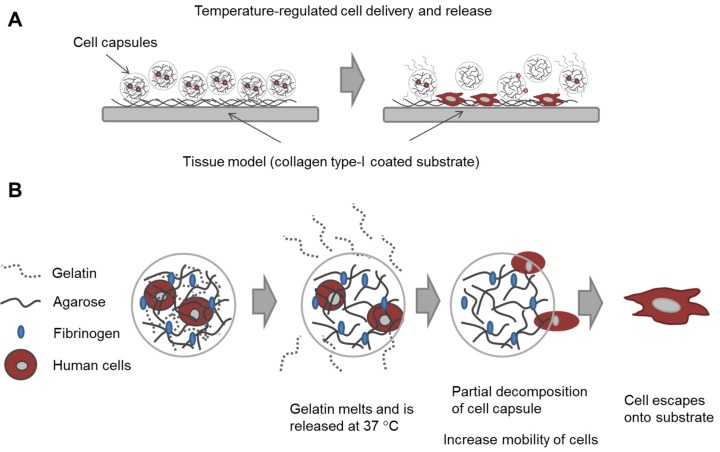
Schematic diagram illustrating (**A**) the concept of cell delivery and release with temperature responsive microcapsules and (**B**) the hypothesized mechanism of cell release from a composite temperature responsive hydrogel comprising two materials with different phase transition temperatures.

In this study, our goal was to build upon and refine the agarose-fibrinogen microcapsules with temperature responsive hydrogel to allow delivery, as well as release of encapsulated cells for future therapeutic application. We tested the hypothesis that the combination of two biomaterials such as low-gelling temperature agarose (gels at <30 °C and melts at >60 °C) and gelatin (melts at >35 °C), which possess different phase transition temperatures from gel to sol, will result in a temperature responsive composite microcapsule that can be used to temporarily encase cells for controlled delivery. For example, at the physiological temperature of 37 °C, low-gelling temperature agarose exists as a solid gel, while gelatin melts, entering the liquid phase and thereby destabilizing the resulting capsule and allowing the encased cells to escape (Figure 1). We verified the hypothesis by fabricating microcapsules of agarose and gelatin, with and without added fibrinogen, and testing these as cell delivery systems that allowed for controlled cell release, using human fibroblast as model cells. We then confirmed the utility of this tool with human umbilical vein endothelial cells (HUVECs) as a model of therapeutic angiogenic cells.

## 2. Results and Discussion

### 2.1. Microencapsulation of Cells

In general, the gelatin incorporated within the agarose capsules served as a temperature switch that controlled the rate of decomposition of the hydrogel capsules. Increasing amounts of gelatin within the agarose–gelatin capsules facilitates capsule decomposition. However, a high concentration of gelatin within the agarose mixture will disrupt the gelation process. The maximum amount of gelatin that could be incorporated into agarose to still form an intact hydrogel capsule was a 1:2 ratio (w/w) of gelatin:agarose. However, for optimal release of cells, a formulation comprising 1% low-gelling temperature agarose, 0.5% gelatin, and 10 mg·mL^−1^ fibrinogen was used for fabrication of cell capsules.

**Figure 2:**
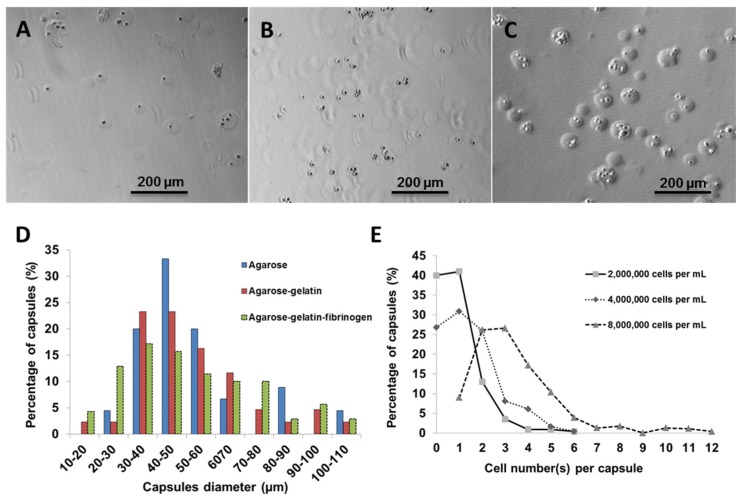
Light micrographs showing cell capsules prepared with various initial cell concentrations (**A**) 2,000,000, (**B**) 4,000,000 and (**C**) 8,000,000 cells·mL^−1^, respectively. (**D**) Size distribution of capsules prepared with various hydrogel formulation. (**E**) Cell number(s) per single capsule (as a function of various initial cell concentrations).

Using fibroblasts as model cells, the number of cells per capsules was optimized by varying the cell number against a fixed volume of hydrogel matrix. Three different cell concentrations (2,000,000; 4,000,000; and 8,000,000 cells·mL^−1^) were used for encapsulation within agarose-gelatin-fibrinogen matrices (Figure 2A–C). The encapsulated cells, also referred to as “cell capsules” were collected and subsequently filtered twice with cell sieves having diameter of 40 µm and 100 µm. With this technique, we are able to prepare cell capsules with a relatively narrow size distribution with ~80% of the cell capsules ranging from 20 to 70 µm in diameter (Figure 2D). It is noticed that cell capsules prepared by more complex hydrogel mixture (*i.e*., the agarose, agarose-gelatin and agarose-gelatin-fibrinogen mixture) will result in broaden of the capsule size distribution curve. The broadening of the capsule size distribution could be explained by the increase of the viscosity of the hydrogel mixture, which leads to the formation of less homogenous droplet during the fabrication process. The cell number(s) per single capsule as a function of various initial cell concentrations were calculated based on cell counting of individual capsules (*n* = 220). Figure 2E shows that the cell density per capsule increased as the initial cell concentration increased. Cell capsules prepared with an initial cell concentration of 2,000,000 cells mL^−1^ had highest population of singly encapsulated cells, while increasing the initial cell concentration to 4,000,000 cells·mL^−1^ and 8,000,000 cells mL^−1^ resulted in a higher proportion of capsules containing multiple cells. Hence, in order to achieve single cell encapsulation, an initial cell concentration of 2,000,000 cells·mL^−1^ was selected for preparation of the cell capsules. However, it is important to note that the proportion of empty capsules increased with a lower initial cell density, due to the Poisson distribution of cells.

### 2.2. Characterization of Cell Capsules

The zeta potential of the hydrogel capsules provides an indicator of the overall surface charge of capsules related to its composition and is a measure of their stability behavior. Hydrogel microcapsules composed of agarose, agarose–gelatin and agarose–gelatin–fibrinogen were therefore characterized by measuring the zeta potential at the different isoelectric points of each of the components. Agarose is a neutral carbohydrate that does not contain ionically charged functional groups. Fibrinogen and type-A gelatin, however, are charged peptides with isoelectric pH values of 4.8 and 8.0, respectively.

We therefore performed zeta potential measurements at pH 4.8, maintaining fibrinogen at a functionally neutral change, and then at pH 8.0 where gelatin had a neutral charge. By holding each component at neutrality, the total surface charge of the hydrogel microcapsules would therefore reflect those of the other components. Results indicates the surface zeta potential of agarose, agarose-gelatin and agarose-gelatin-fibrinogen microcapsules measured at pH 4.8 were 1.89 ± 0.28 mV, 22.9 ± 0.36 mV and 24.6 ± 0.95 mV, respectively; while zeta potential measured at pH 8.0 were 2.42 ± 0.13 mV, −3.01 ± 0.26 mV and −14.73 ± 1.41 mV, respectively (Table 1). The zeta potential studies showed that the addition of gelatin and fibrinogen increased the zeta potential of the microcapsules from 0 mV towards ±30 mV. This shows that addition of peptide components increased the capacity of the microcapsules to exist as stable individual units and not coagulate. The three materials all displayed significantly differing zeta potentials (GLM *p* ≤ 0.01). The only material that did not have a significantly different zeta potential at pH 8 compared to pH 4.2 was agarose (GLM *p* ≤ 0.01, Tukey *p* ≤ 0.05). 

**Table 1:** Summary of zeta potential of different hydrogel microcapsules.

Capsule Component(s)	pH 4.8	pH 8.0
Agarose	Agarose (neutral)	Agarose (neutral)
	Zeta potential 1.89 ± 0.28 mV	Zeta potential 2.42 ± 0.13 mV
Agarose–gelatin	Agarose (neutral)	Agarose (neutral)
	Gelatin (+ve)	Gelatin (neutral)
	Zeta potential 22.9 ± 0.36 mV	Zeta potential −3.01 ± 0.26 mV
Agarose–gelatin–fibrinogen	Agarose (neutral)	Agarose (neutral)
	Gelatin (+ve)	Gelatin (neutral)
	Fibrinogen (neutral)	Fibrinogen (−ve)
	Zeta potential 24.6 ± 0.95 mV	Zeta potential −14.73 ± 1.41 mV

FTIR spectroscopy was used to analyze the composition of the hydrogel microcapsules. The structural spectral features of gelatin such as α-helix and β-sheet can be inferred from amide I and amide II bands in the region of 1700–1600 and 1600–1500 cm^−1^, while the structural features of agarose, such as pyranose, can be inferred from absorption bands at 1200–970 cm^−1^ due to C–C and C–O stretching within the pyranoid ring and to C–O–C stretching of glycosidic bonds. The FTIR spectra of agarose microcapsules and agarose–gelatin microcapsules demonstrated the successful doping of agarose with gelatin, forming agarose–gelatin hybrid microcapsules (Figure S1). The presence of α-helix and β-sheet structures in the FTIR spectra suggested that the secondary structure of the gelatin within the microcapsules remained similar to that of the native macromolecules. However, FTIR results can only demonstrate gelatin structural integrity at the level of secondary conformation, while the structural integrity of the tertiary and quaternary structure of the gelatin within the agarose–gelatin microcapsules remained unclear.

### 2.3. Viability of the Encapsulated Cells

The viability of the encapsulated human fibroblast cells and HUVECs was assessed with a LIVE/DEAD^®^ staining kit composed of calcein AM and ethidium homodimer (EthD-1) dyes. Calcein AM measures the intracellular activities of viable cells producing a green fluorescence signal (ex/em ~495 nm/~515 nm), while EthD-1 passively penetrates into dead cells with disrupted plasma membrane and binds to nucleic acids, producing a red fluorescent signal (ex/em ~495 nm/~635 nm).

**Figure 3:**
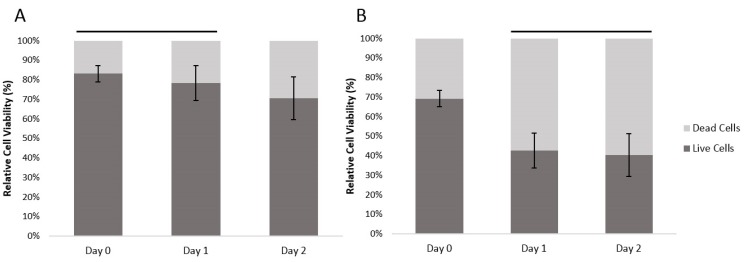
The relatively cell viability of (**A**) encapsulated human fibroblast cells and (**B**) encapsulated HUVECs over time, within the agarose–gelatin–fibrinogen microcapsules. Time points with a connecting line over their bars were not found to be significantly different. Bars not connected by lines were found to be significantly different (GLM using Tukey *post hoc*, *p* ≤ 0.05).

With human fibroblasts, the encapsulation process resulted in 19.9% dead cells (at Day 0). With HUVECs, which are more sensitive cells, the encapsulation resulted in 30.1% dead cells. Between Days 0 and 1, the ratio of dead fibroblasts had increased by 4.9% and from Day 1 to Day 2, the ratio of dead cells increased a further 7.8%. For the HUVEC cells, the ratio of dead cells increased by 26.6% between Day 0 and Day 1, and there was a further 2.2% increase in the ratio of dead HUVEC cells between Day 1 and 2 (Figure 3). There was a significant difference between cell viability of the two different cell types (General linear model/GLM, *p* ≤ 0.01). Both cell types had significant differences between time points (GLM, *p* ≤ 0.01), the dynamics of the overtime change differed between cell types. Fibroblast survival did not show a significant difference between Day 0 and Day 1; at Day 2 there was, however, a significantly smaller ratio of live cells. The HUVEC cells on the other hand, showed a significant loss of live cells between Day 0 and Day 1. However, between Day 1 and 2 there was no significant further loss.

The results show that our developed method can reliably encapsulate viable fibroblast and HUVECs. The absolute cell viability, however, is dependent upon the cell type. The encapsulation process itself consistently killed an initial proportion of cells. This is most likely due to the shear forces generated during the encapsulation. Cell death amongst the fibroblasts took 48 hours as would be expected by a more resilient cell type. The more delicate, damaged HUVEC cells continued to die during Day 1. By Day 2, the HUVECs numbers stabilized. 

### 2.4. Temperature Regulated Decomposition of Cell Capsules

**Figure 4:**
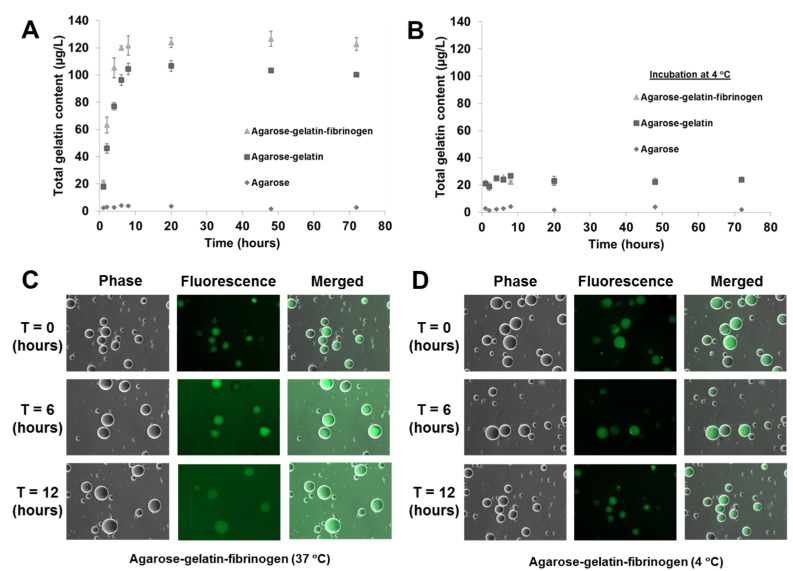
Decomposition kinetics of hydrogel microcapsules measured by release of gelatin (**A**) at 37 °C and (**B**) control at 4 °C as a function of time. (**C**,**D**) Optical images showing the decomposition and release of fluorescent-labeled gelatin into the suspended PBS solution, causing an increase of background fluorescence intensity at 37 °C, but not at 4 °C.

As previously mentioned, the temperature responsive microcapsules were designed based on our hypothesis that doping of agarose hydrogels that are solid from 30 to 60 °C with gelatin that melts <35 °C) can facilitate the decomposition of the microcapsules at a physiological temperature of 37 °C, due to the gelatin melting and diffusing out of the microcapsules. A decomposition study of agarose only, agarose–gelatin and agarose–gelatin–fibrinogen microcapsules was performed to verify the hypothesis by quantifying the release of gelatin over time, for a total of 72 h when the microcapsules in PBS were incubated at 37 °C. The released gelatin was quantified by performing a total protein assay. The decomposition kinetics as a function of gelatin release is shown in Figure 4A,B. Agarose only microcapsules were used as controls. The results obtained showed that at 37 °C, the decomposition and release of melted gelatin from the microcapsules was completed within the first 8 h, followed by a constant steady state observed from 8 to 72 h where no further release of gelatin occurred. In contrast, microcapsules incubated at 4 °C showed insignificant release of gelatin over the entire 72 h, confirming that the decomposition mechanism was triggered by the elevated, physiological temperature. Both the agarose–gelatin and agarose–gelatin–fibrinogen microcapsules showed a similar release profile, although the agarose–gelatin–fibrinogen microcapsules had a larger signal amplitude. This can be explained by the relatively stable encapsulation of fibrinogen within hydrogel microcapsules similar to previously reported [4], whereby the releasing only small amount of fibrinogen contributed to the total protein content. The decomposition of agarose–gelatin–fibrinogen microcapsules was further confirmed by observation of fluorescent-labeled gelatin released when incubated at 37 °C, while release of fluorescent-labeled gelatin was not observed when incubated at 4 °C. The released fluorescent-labeled gelatin into the buffer resulted in an overall increase of background fluorescence intensity of the PBS solution (Figure 4C,D).

### 2.5. Delivery of Encapsulated Human Fibroblasts 

**Figure 5:**
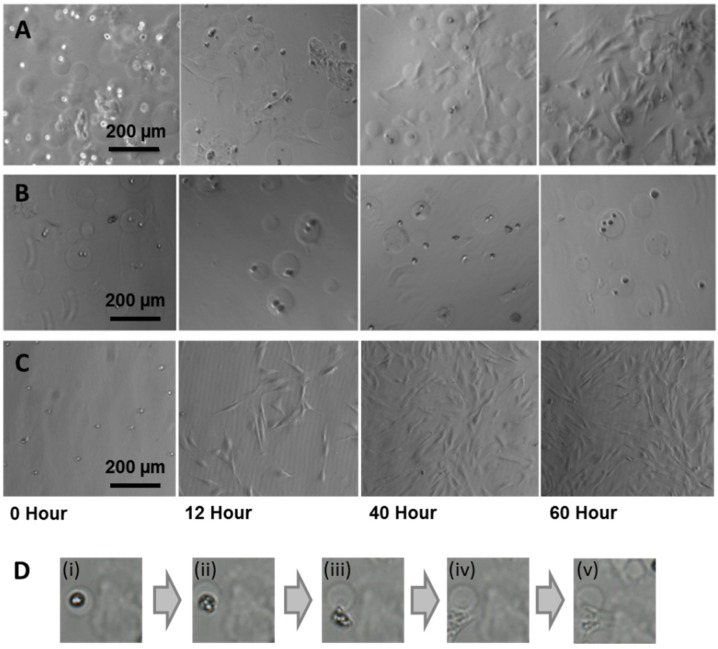
Optical micrographs showing the release of human fibroblast cells onto collagen coated tissue culture dishes from microcapsules composed of (**A**) agarose–gelatin–fibrinogen, (**B**) agarose only negative controls and (**C**) non-encapsulated fibroblast controls. (**D**) Sequence of images (i) to (v) with duration of ~1.5 h showing the release of a human fibroblast cell from an agarose–gelatin–fibrinogen capsule onto the culture substratum.

Temperature regulated cell release was detected as a decrease in the number of cells (with rounded morphology) found within microcapsules that corresponded to an increase in the number of released cells, visualized as cells that had attached and spread on the collagen substratum (Figure 5A). Control capsules comprising agarose alone did not result in cell release (Figure 5B) and non-encapsulated fibroblasts control (Figure 5C). This confirmed our hypothesis that the gelatin was needed as a temperature responsive agent by facilitating decomposition of the capsule at physiological temperatures.

The captured images of the cell release process further revealed that in conjunction with the capsule decomposition, cell release was initiated by an increase in cell mobility as the hydrogel capsules become partially decomposed due to the melting and dissipation of the gelatin triggered by temperature (Figure 5Di). Next, the cells emerged from weakened spots within the hydrogel capsule (Figure 5Dii), followed by attachment and spreading on the culture substratum (Figure 5Diii–v).

**Figure 6:**
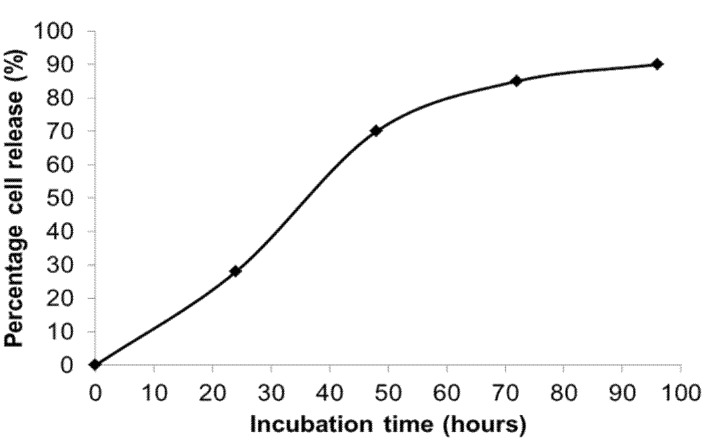
Kinetics of cell release from temperature responsive microcapsules comprising 1% low melting agarose, 0.1% to 0.5% gelatin, and 10 mg·mL^−1^ fibrinogen, showing that 28% of cells were released within 24 h, and 70% cells were released by 48 h.

The kinetics of cell delivery defined by the percentage of encapsulated cell delivered/released from hydrogel capsules was measured by counting the initial number of encapsulated cells (inside hydrogel capsule with rounded morphology) and released cells as a function of time. For microcapsules comprising 1% low melting agarose, 0.5% gelatin, and 10 mg mL^−1^ fibrinogen, 28% of the cells were released within 24 h of incubation at 37 °C. By 48 h, 70% of cells were released (Figure 6).

### 2.6. Delivery of HUVECs 

**Figure 7:**
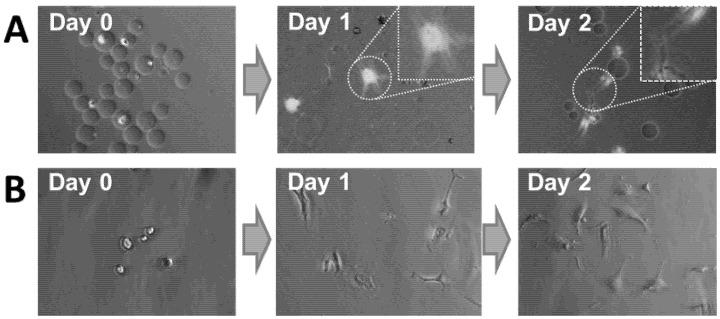
(**A**) Micrographs of HUVECs within agarose–gelatin–fibrinogen microcapsules at Day 0 and after their release. On Day 1, released HUVECs are seen attached and spread after their release onto gelatin-coated tissue culture plastic. By Day 2, several cells have migrated and have aligned themselves into a cord-like structure that is typical of HUVEC *in vitro* tubulogenesis behavior [27]. (**B**) Non-encapsulated HUVEVs control.

We have shown using human fibroblasts as a model cell line that cells can be very simply and effectively encapsulated within hydrogel microcapsules and subsequently released onto gelatin coated tissue culture plates. With a view to potential clinical application, we also showed that the methodologies developed were also applicable for endothelial cells, e.g., for revascularization of ischemic tissues such as heart muscle after a myocardial infraction, while HUVECs were more delicate and had a higher rate of cell death due to the encapsulation process (as shown in Figure 4). We observed that the released HUVECs retained their ability to migrate on the gelatin substratum to form cord-like structures that are precursors to the *in vitro* tubulogenesis (Figure 7), a behavior typical of HUVEC on gelatin [27]. This showed that the encapsulation did not adversely alter the behavior of the encapsulated cells, an important criterion for potential clinical application.

## 3. Experimental Section 

### 3.1. Materials

Low-gelling temperature agarose (A9045), gelatin (300 bloom from porcine skin; G2500), fibrinogen (F8630), mineral oil (M8410), Endothelial Cell Growth Supplement (ECGS; E2759), heparin (H3149), Span-80 non-ionic surfactant (S6760) and fluorescein isothiocyanate (F3651) were purchased from Sigma-Aldrich (St. Louis, MO, USA). DEAD/LIVE^®^ staining kit was purchased from Molecular Probes-Life Technology (Stockholm, Sweden). Dulbecco’s Modified Eagle Medium (DMEM), M199-medium and GlutaMAX™ were purchased from Gibco^®^-Life Technology (Stockholm, Sweden).

### 3.2. Cells 

Human fibroblasts (CRL-2522; ATCC, Germany) were cultured in DMEM containing 10% fetal bovine serum, 100 U·mL^−1^ penicillin and 100 µg·mL^−1^ streptomycin. Human umbilical vein endothelial cells (HUVECs) were cultured on gelatin-coated cell culture flask in M199-medium supplemented with 50 µg·mL^−1^ ECGS, GlutaMAX™ 1X, heparin 50 µg·mL^−1^, 5% fetal bovine serum, 100 U mL^−1^ penicillin and 100 µg·mL^−1^ streptomycin. HUVECs were immortalized using the human papilloma virus E6E7 proteins, which are believed to restore telomere length to primary human cells as previously published [28,29]. For this study, they were transfected with lentiviral vectors containing eGFP (LV/eGFP) to produce cells that fluoresced green for easy tracking after encapsulation. VSV-G-pseudotyped LVs were generated by transient transfection of 293T cells with three plasmids (LV plasmid construct, packaging plasmid pCMVΔ8.91 and the VSV-G envelope-coding plasmid pMD.G) using the TransIT-LT1 transfection reagent (Mirus Nio LLC, Madison, WI, USA). The concentrated virus was suspended in serum-free DMEM medium and stored at −80 °C until use. The viral titers were then determined by flow cytometric analysis using a FACS Calibur (BD Biosciences). HUVECs were grown in DMEM supplemented with 10% FCS. They were then transduced with LV/eGFP (M.O.I. = 5) and then enriched by 0.5 μg·mL^−1^ of puromycin selection.

### 3.3. Cell Encapsulation

**Figure 8:**
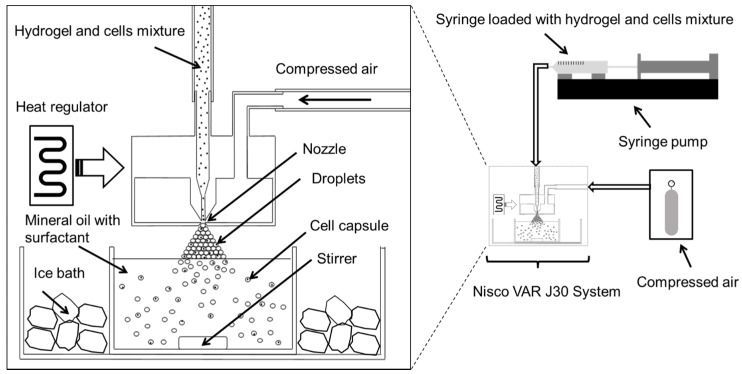
Schematic diagram illustrating the high throughput preparation of cell microcapsules using the Nisco VAR J30 System.

For encapsulation, 2.0 × 10^6^ to 8.0 × 10^6^ cells were re-suspended in approximately 500 μL of medium, DMEM for fibroblast or M199 for HUVECs, then mixed with 500 μL of the encapsulation hydrogel, which comprised a mixture of 2% low-gelling temperature agarose, 1% gelatin and 10 mg·mL^−1^ fibrinogen in medium at 40 °C. Control experiments included encapsulation of cells in agarose only, and empty agarose–gelatin capsules. A diagram showing our cell encapsulation system is given in Figure 8. In brief, cell-hydrogel mixtures were loaded into a syringe and extruded through a regulated nozzle of 25 μm in diameter, warmed at constant temperature of 40 °C. The flow rate was controlled by use of a syringe pump that was set at a flow rate of 0.3 mL min^−1^ that created a spray of microdroplets. The air pressure was set at 350 mBar. The microdroplets were collected in an ice-cooled mineral oil bath containing 0.5% Span-80. The bath was stirred for 5 min allowing the solidification of the hydrogel droplets. The solution mixture containing encapsulated cells was then transferred to a 50 mL centrifugation tube. They were then washed by addition of 15 mL of medium followed by centrifugation at 350 r.c.f. at 4° for 10 min. The bottom, aqueous fraction containing the cell capsules was collected and transferred to a 15 mL centrifuge tube. The cell capsules were then washed another two times with medium followed by centrifugation (350 r.c.f., 10 min) at room temperature. Finally, the cell capsules were re-suspended in medium and filtered through a 100 μm cell strainer followed by a 40 μm cell strainer where the captured cells where used for further experiments.

### 3.4. Decomposition Study of the Temperature Responsive Hydrogel Microcapsules

Thermal destabilization or decomposition of the temperature responsive hydrogel microcapsules was achieved by incubation of 5 mL of the hydrogel microcapsule suspension at 37 °C in 10 mM phosphate buffer (pH 7.4) over a period of 72 h. The thermal decomposition was measured as a function of melted gelatin released from hydrogel microcapsules at 37 °C. Control experiments were performed by incubating the hydrogel microcapsules at 4 °C under the same conditions. Supernatants were collected at various time intervals and the amounts of gelatin released into the supernatants were quantified by using a BCA total protein assay kit. The presence of gelatin resulted in the formation of a water-soluble complex with an optical density at 562 nm that was recorded with a UV-Vis spectrophotometer (Shimadzu UV-2450, Kyoto, Japan).

### 3.5. Cell Delivery and Release from Capsules

Optical and fluorescence microscopy images were recorded on a light microscope (Zeiss Axio Vert.A1, Oberkochen, Germany) connected to a CCD color digital camera (AxioCam Cm, Zeiss, Germany). Images of cells within their capsules were captured and analyzed using the accompanying imaging software (Zen2012 blue edition, Zeiss, Germany).

Temperature regulated cell delivery and cell release was performed by placing cell capsules composed of microencapsulated human fibroblasts onto surface of type I collagen coated tissue culture plates incubated at 37 °C. To follow the cell delivery and release behavior, the cell capsules were monitored by a live image capturing system with pictures taken every 15 min. Live video capture of the differential rates of cell escape from their microcapsules was performed using a live image capturing system, the JULI-Smart fluorescent cell imager (Ruskinn, ME, USA) with images taken every 15 min. The viability of the encapsulated cells was visualized with a LIVE/DEAD^®^ staining kit composed of probing dyes of calcein AM for viable cells and ethidium homodimer (EthD-1) for dead cells. For statistical analysis of survival rates the general linear model (GLM) with Tukey *post hoc* tests were used.

### 3.6. Zeta Potentials Analysis

The zeta potential (surface charge) of the hydrogel microcapsules was measured using a Zetasizer Nano ZS90 (Malvern Instruments Ltd., Worcestershire, UK), based on the laser doppler micro-electrophoresis principle. The electrophoretic mobility (μ) was converted to the zeta-potential (ζ) by using the Smoluchowski relation ζ = μη/ε, where η and ε are the viscosity and permittivity of the solution, respectively. One milliliter of suspended hydrogel microcapsules in 10 mM phosphate buffer with various pH values were loaded into a zeta potential measuring cell. The measurements were performed at 25 °C and the mean zeta potential values were calculated by taking an average of 3 repeated measurements.

### 3.7. Fourier Transform Infrared Study

Fourier Transform Infrared (FTIR) characterization of microcapsules was performed using a VERTEX 70 instrument (Bruker, WA, USA) equipped with a germanium attenuated total reflectance (ATR) sample cell. Hydrogel microcapsule suspensions were dropped onto the surface of the ATR cell and FTIR spectra were recorded in the frequency region of 600–4000 cm^−1^ with a resolution of 4 cm^−1^ and run for 100 cycles.

## 4. Conclusions 

We have shown that the phase transition properties of two biomaterials—agarose and gelatin—can be exploited for fabrication of temperature responsive hydrogel microcapsules for the encapsulation, delivery and release of human fibroblasts and HUVECs. Cell release was facilitated by the dissolution of the thermally-sensitive biopolymer, in this case, gelatin, that was incorporated into the agarose capsules as a cell-release agent. At physiological temperature, its melting weakened the capsules allowing escape of the cells into the target environment. The cell capsules were fabricated using a robust, high throughput microencapsulator. The use of a microencapsulator over the conventional reverse emulsion system allowed for control over cell number and capsule size, which are necessary for protocol standardization for future clinical application. We will, however, need to augment the composition of the microcapsules and optimize the numbers of cells per capsule prior to *in vivo* animal studies, as we explore the potential of these cell capsules for future clinical application. 

## Supplementary Files

Supplementary File 1
