# Correction: W.C. Mak, et al. Controlled Delivery of Human Cells by Temperature Responsive Microcapsules. *J. Funct. Biomater.* 2015, *6*, 439–453

**DOI:** 10.3390/jfb9020026

**Published:** 2018-03-21

**Authors:** W.C. Mak, K. Olesen, P. Sivlér, C.J. Lee, I. Moreno-Jimenez, J. Edin, D. Courtman, M. Skog, M. Griffith

**Affiliations:** 1 Department of Clinical and Experimental Medicine, Linköping University, SE58185 Linköping, Sweden; kim.olesen@liu.se (K.O.); petter.sivler@s2m.se (P.S.); chyanjang@gmail.com (C.J.L.); inesmorenojimenez@gmail.com (I.M.-J.); joel.edin@liu.se (J.E.); marten.skog@liu.se (M.S.); may.griffith@liu.se (M.G.); 2 Biosensors and Bioelectronics Centre, Department of Physics, Chemistry and Biology, Linkӧping University, SE58183 Linköping, Sweden; 3 Bone & Joint Research Group, Stem Cells & Regeneration Institute of Developmental Sciences, Southampton General Hospital, Southampton, Hampshire SO16 6YD, UK; 4 Regenerative Medicine Program, Ottawa Hospital Research Institute, Ottawa, ON K1H 8L6, Canada; dcourtman@ohri.ca

Recently, we found a mistake in Figure 4D in our previously published paper [1], which we would like to correct. In brief, the images for Figure 4D at time 12 h are incorrect and should be replaced as shown below (Figure 1). This amendment does not influence the conclusions drawn in the paper. We wish to apologize for any inconvenience that this may have caused.

## Figures and Tables

**Figure 1:**
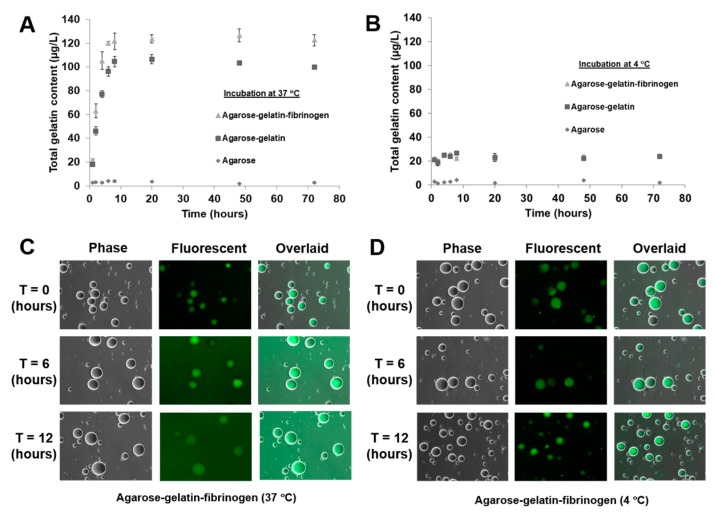
Decomposition kinetics of hydrogel microcapsules measured by release of gelatin (**A**) at 37 °C and (**B**) control at 4 °C as a function of time. (**C**,**D**) Optical images showing the decomposition and release of fluorescent-labeled gelatin into the suspended PBS solution, causing an increase of background fluorescence intensity at 37 °C, but not at 4 °C.
