# Supplementary figures and images for: Controlled Delivery of Human Cells by Temperature Responsive Microcapsules
Source: J Funct Biomater. 2015 Jun 18;6(2):439–53. doi: 10.3390/jfb6020439 (PMC4493523; doi:10.3390/jfb6020439)

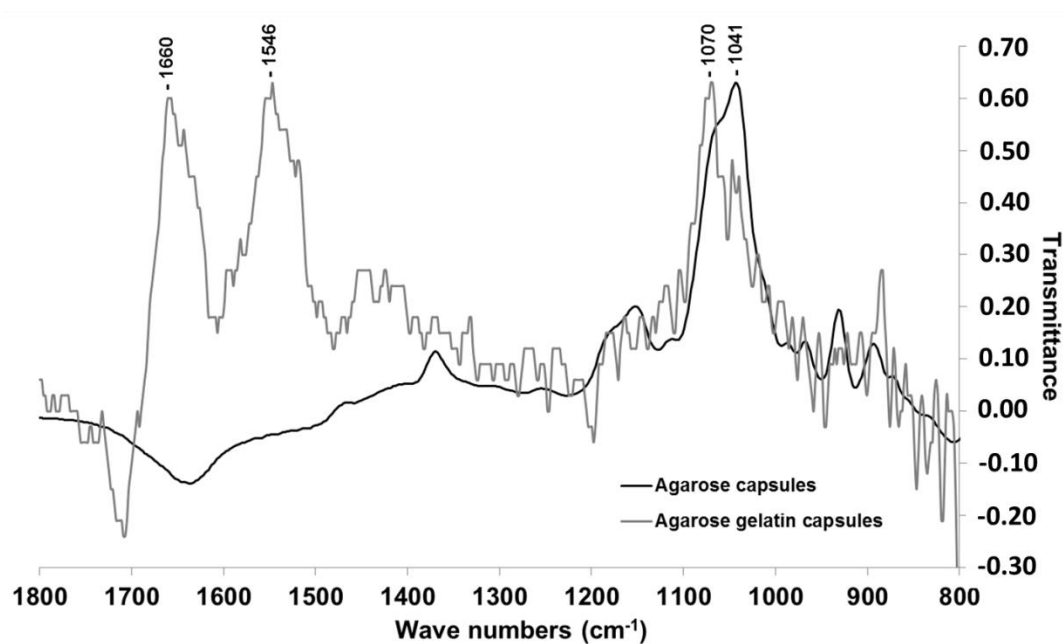

**Figure S1.** FTIR spectra of agarose microcapsules and agarose-gelatin microcapsules.

Supplement: Supplementary File 1 [file jfb-06-00439-s001.pdf]
